# Pre-procedural left atrial strain and clinical features for predicting recurrence after first catheter ablation of persistent atrial fibrillation: a prospective cohort study

**DOI:** 10.3389/fcvm.2026.1764723

**Published:** 2026-02-09

**Authors:** Qunfang Tu, Dongping Xiao

**Affiliations:** 1 Department of Emergency, The First Hospital of Nanchang, Nanchang City, Jiangxi, China; 2 Department of Cardiology, The First Hospital of Nanchang, Nanchang City, Jiangxi, China

**Keywords:** atrial fibrillation recurrence, catheter ablation, peak atrial longitudinal strain, persistent atrial fibrillation, risk prediction model

## Abstract

**Background:**

Recurrence after the first ablation for persistent atrial fibrillation (AF) is common. Existing clinical and structural prediction models have limited performance. Peak atrial longitudinal strain (PALS) quantifies left atrial function and may improve risk stratification.

**Objective:**

To develop and validate a 12-month recurrence prediction model for persistent AF by integrating PALS with clinical features, and to evaluate its performance and clinical utility.

**Methods:**

Single-center prospective cohort; of 629 patients screened, 429 were enrolled, and 410 were included in the final analysis after exclusions for image quality and loss during the blanking period. Pre-procedural PALS was measured with standardized quality control. The outcome was the first recurrence (>30 s) between the end of the 90-day blanking period and 12 months. Cox models with restricted cubic splines assessed the dose–response. Model performance (C-index, time-dependent AUC, Brier score, calibration) was compared among “Clinical-only”, “Clinical + PALS”, and “Clinical + LAVI” models. Reclassification (NRI/IDI) and clinical net benefit (decision curve analysis) were evaluated, along with sensitivity and interaction analyses.

**Results:**

PALS independently predicted recurrence (per 1% decrease: HR 1.06, 95% CI 1.03–1.10; *p* < 0.001), with excellent reproducibility (ICC intraobserver 0.91–0.93, interobserver 0.87–0.91). Adding PALS to the Clinical-only model improved discrimination (C-index 0.74 vs. 0.66; ΔAUC 0.08, *p* < 0.001) and calibration (*α* = −0.02, *β* = 0.94). Reclassification improved (NRI 0.43; IDI 0.07; *p* < 0.01). The Clinical + PALS model provided greater net benefit across thresholds of 10%–30% (max ΔNB 0.06 at 24%). The PALS effect was consistent across acquisition rhythms (*p*-interaction = 0.417). Adding LAVI yielded more modest improvement (C-index 0.69; ΔAUC 0.03, *p* = 0.084).

**Conclusions:**

Pre-procedural PALS significantly improves individualized prediction of 12-month recurrence after first ablation for persistent AF when combined with clinical features. The model shows robust performance and clinical net benefit, supporting PALS as a core functional metric in pre-procedural assessment.

## 1 Introduction

Catheter ablation is a cornerstone therapy for persistent atrial fibrillation (AF), aimed at improving symptoms and restoring rhythm control (1, 2). However, mid-term recurrence after a first ablation procedure remains common, imposing significant burdens on patients and healthcare systems through repeat procedures, intensified monitoring, and continued medical therapy (3). Effective post-procedural management, therefore, critically depends on preprocedural, individualized risk stratification to rationally allocate follow-up resources and guide timely reintervention.

Existing clinical prediction models for AF recurrence predominantly rely on demographic factors, comorbidities, and imaging measures of left atrial (LA) *structure*, such as the left atrial volume index (LAVI), in conjunction with left ventricular function (4). While useful, these models often demonstrate limited discriminative ability and calibration, which reduces their reliability in supporting threshold-based clinical decisions at the individual patient level (5). This limitation may stem from an incomplete characterization of the atrial substrate; persistent AF is a progressive disease driven by complex structural and electrical remodeling, where functional impairment and fibrosis often precede or exceed mere geometric dilation (1, 6). Consequently, a functional metric that directly quantifies LA reservoir performance and fibrotic burden could capture a more proximate pathophysiological pathway to recurrence.

Peak atrial longitudinal strain (PALS), derived from two-dimensional speckle-tracking echocardiography, provides just such a measure. It quantifies the LA reservoir function during ventricular systole, integrating information on LA compliance, wall stress, and myofiber orientation (7). A diminished PALS reflects restricted LA deformation, a marker of diffuse interstitial fibrosis and electromechanical dysfunction, a substrate known to promote AF persistence and recurrence after ablation (8, 9). As a widely accessible, non-invasive tool, PALS holds significant potential as a core functional metric to enhance pre-ablation risk assessment (10).

Despite this promise, systematic evidence for PALS in the specific context of first-time ablation for persistent AF remains underdeveloped. Many prior studies have been retrospective, included mixed AF types, or employed non-standardized PALS acquisition without rigorous quality control (11, 12). Methodologically, there is a paucity of studies that prospectively evaluate the *incremental* predictive value of PALS over established clinical features within a “purely preprocedural” framework (i.e., using only variables known before the ablation procedure is performed). Crucially, comprehensive validation, including calibration assessment, reclassification metrics, and, most importantly, an evaluation of clinical utility via decision curve analysis across relevant risk thresholds, is frequently lacking (13). Furthermore, it remains unclear whether the predictive value of PALS is robust across different acquisition rhythms (sinus rhythm vs. AF), and direct, methodologically consistent comparisons demonstrating its superiority over traditional structural metrics, such as LAVI, are scarce (14).

To address these gaps, we conducted a prospective cohort study with the primary aim of developing and rigorously validating a preprocedural prediction model for 12-month recurrence after first-time catheter ablation for persistent AF. We hypothesized that integrating standardized PALS measurements with clinical features would yield a model with superior discrimination, calibration, and reclassification compared to a clinical-only model, and that this model would provide a tangible net benefit for clinical decision-making within reasonable probability thresholds. To test this, we systematically compared three models (“clinical-only,” “clinical + PALS,” and “clinical + LAVI”) using a time-to-event framework from the end of the blanking period, incorporating internal validation, assessment of rhythm stability, and a full spectrum of performance metrics, including decision curve analysis.

## 2 Materials and methods

### 2.1 Study design and ethics

This was a single-center, prospective, observational cohort study. The study protocol was approved by the Institutional Review Board/Ethics Committee of Nanchang First Hospital, Jiangxi (Approval Number: KY2021035). All participants provided written informed consent before enrollment. The study period was from June 1, 2021, to March 31, 2025. The enrollment window closed on March 31, 2024, to ensure a minimum of 12 months of potential follow-up for all participants.

### 2.2 Study population and screening

Between June 1, 2021, and March 31, 2024, we screened 629 consecutive patients with persistent atrial fibrillation (AF) who were scheduled for first-time catheter ablation at our center. After applying the pre-specified inclusion and exclusion criteria, 200 patients were excluded: 172 did not meet eligibility criteria (see the detailed breakdown in Supplementary Table S1), and 28 declined participation or withdrew consent. Thus, 429 patients provided written informed consent and were enrolled in the prospective cohort.

***Inclusion Criteria*:** Patients were eligible if they were aged 18–85 years, had a confirmed diagnosis of persistent AF (continuous episode ≥7 days or requiring cardioversion for termination), were scheduled for a first-time left atrial catheter ablation, and could complete standardized transthoracic echocardiography and laboratory assessments within 14 days before the procedure while adhering to the follow-up protocol.

***Exclusion Criteria:*** We excluded patients with any of the following: prior left atrial catheter ablation or surgical maze procedure; cardiac surgery or percutaneous coronary intervention within the preceding 3 months; significant rheumatic mitral valve disease or mechanical prosthetic valves; complex congenital heart disease; active myocarditis or restrictive/infiltrative cardiomyopathies; life expectancy <1 year; pregnancy; inadequate echocardiographic image quality for strain analysis; or withdrawal of consent.

### 2.3 Sample size justification

The primary aim was to develop a multivariable prediction model. The core model for evaluating the incremental value of PALS was pre-specified to include PALS plus 7 clinical covariates [age, sex, AF duration, body mass index (BMI), hypertension, diabetes, and estimated glomerular filtration rate (eGFR)], totaling 8 parameters. Adhering to the rule of thumb requiring a minimum of 15 events per variable (EPV), we needed at least 120 events. Based on published data and institutional experience, we conservatively estimated a 12-month post-blanking recurrence rate of 35%. Thus, the minimum required sample size was 120/0.35 ≈ 343 patients. Accounting for an estimated 5% loss to follow-up, the target enrollment was set at 410 patients (343/0.95 ≈ 361, rounded up to ensure robustness and allow for additional covariates in secondary analyses).

### 2.4 Study workflow, timeline, and outcome definition

The day of the first catheter ablation procedure was defined as the Index date. All baseline assessments were completed within the 14 days preceding the Index. The blanking period was defined as the first 90 days post-ablation. The primary outcome was the first documented recurrence of any atrial arrhythmia (AF, atrial flutter, or atrial tachycardia lasting ≥30 s) occurring after the blanking period and within 12 months of the Index date. A 12-lead electrocardiogram, a 24 h Holter monitor, a patch monitor, or remote transmission from an implanted cardiac device confirmed recurrence. All potential events were adjudicated by two independent electrophysiologists, who were blinded to PALS measurements, with a third senior expert resolving any disagreements.

### 2.5 Follow-up and monitoring protocol

Routine follow-up visits were scheduled at 1, 3, 6, and 12 months after the ablation. Systematic rhythm assessment included 24 h Holter monitoring at 3, 6, and 12 months. Patients were provided with event recorders for symptom-triggered recordings. For those with implanted cardiac electronic devices, remote monitoring was activated. Loss to follow-up was defined as voluntary withdrawal or loss of contact; the last confirmed contact date was used for censoring in time-to-event analysis.

### 2.6 Pre-procedural echocardiography and PALS measurement

#### 2.6.1 Image acquisition & analysis

Echocardiography was performed using a GE Vivid E95 ultrasound system equipped with an M5Sc phased-array transducer. Images were acquired at a frame rate of 60–90 frames per second. Apical four-chamber and two-chamber views were obtained with careful optimization of the left atrium. For patients in sinus rhythm, three consecutive cardiac cycles were stored; for those in atrial fibrillation at the time of acquisition, ten consecutive cycles were recorded. Offline speckle-tracking analysis was performed using dedicated software (EchoPAC version 203, GE Healthcare). Global PALS was defined as the peak positive strain value occurring at left ventricular end-systole, calculated as the average of the values obtained from the four-chamber and two-chamber views (10).

#### 2.6.2 Quality control & reproducibility

Two blinded, certified analysts performed measurements. Cases were excluded if tracking quality was scored <80% or if ≥1 segment was untrackable. Reproducibility was assessed in 50 randomly selected cases for intra- (4-week interval) and inter-observer variability. Intraclass correlation coefficients (ICC, two-way random, single measures) and Bland-Altman limits of agreement were calculated, with pre-specified quality thresholds of ICC ≥ 0.80 and absolute bias ≤1.5%.

#### 2.6.3 Concomitant measurements

Left atrial volume was measured at end-systole using the biplane disk method and indexed to body surface area (LAVI, mL/m^2^) using the Du Bois formula (11). Left ventricular ejection fraction (LVEF) was calculated via the biplane Simpson method.

### 2.7 Clinical and procedural data

Baseline demographic and clinical data were collected prospectively. Serum creatinine was measured, and eGFR was calculated using the 2021 CKD-EPI equation (12). All catheter ablation procedures had pulmonary vein isolation as a baseline, with adjunctive substrate modification at the operator's discretion. Antiarrhythmic drug use during the blanking period was permitted. To preserve the pre-procedural nature of the prediction model, ablation strategy and post-blanking antiarrhythmic drug use were not included in the core models but were used in sensitivity analyses.

### 2.8 Bias control and data management

A comprehensive data management plan was implemented using the REDCap electronic data capture system. Key biases were mitigated by: (1) blinding echocardiographic analysts to outcomes and clinical data, (2) blinding outcome adjudicators to imaging data and model predictions, (3) applying a standardized follow-up and monitoring protocol for all participants to minimize detection bias, and (4) maintaining separation between analysis workstations and the clinical database.

### 2.9 Statistical analysis

Analyses were conducted using R v4.3.2. A two-sided *p*-value < 0.05 denotes statistical significance.

#### 2.9.1 Model development and primary analysis

For comparative evaluation, three prediction models were developed. The baseline Clinical Model included pre-specified clinical covariates: age, sex, atrial fibrillation duration, body mass index, hypertension, diabetes, and estimated glomerular filtration rate. To assess the incremental value of functional and structural imaging, two enhanced models were constructed: the Clinical + PALS Model, which added peak atrial longitudinal strain to the clinical set, and the Clinical + LAVI Model, which added the left atrial volume index as a structural comparator.

**Figure 1:**
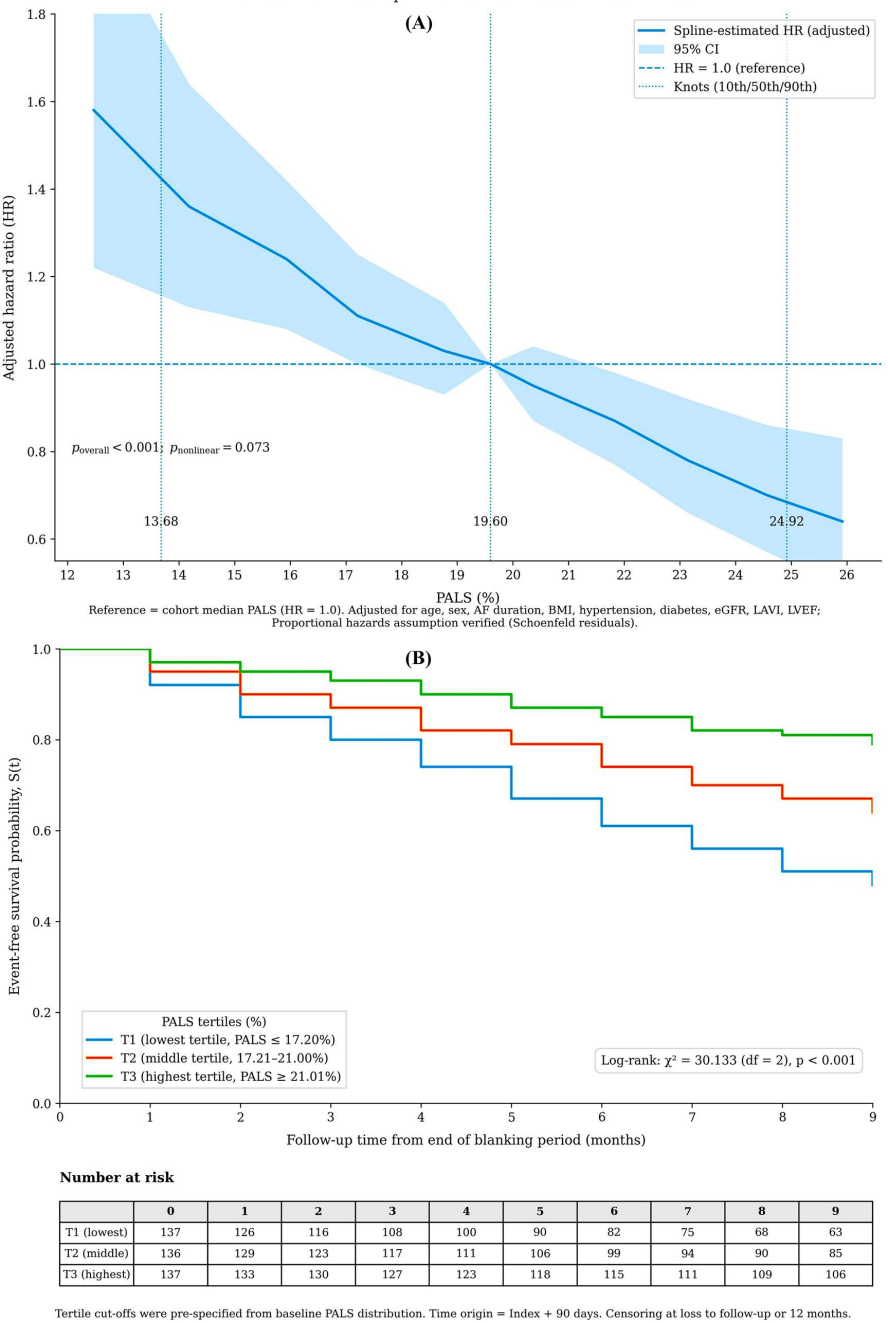
Association of pre-procedural peak atrial longitudinal strain (PALS) with recurrence risk. **(A)** Adjusted restricted cubic spline of continuous PALS (%) vs. hazard ratio. The model was adjusted for clinical and imaging covariates. The dashed line indicates the reference at median PALS (19.6%); *p* for overall association. (**B**) Kaplan-Meier curves stratified by PALS tertiles (log-rank *p* < 0.001).

Continuous predictors were modeled as continuous. Non-linearity was assessed for each continuous variable (including PALS, LAVI, age, AF duration, BMI, eGFR, and LVEF) using likelihood ratio tests comparing a model with a restricted cubic spline term (with knots at the 10th, 50th, and 90th percentiles) to a model with a linear term. A *p*-value < 0.10 for the spline term was pre-specified as evidence of non-linearity, justifying the use of a spline. This formal testing confirmed a linear relationship for most covariates but indicated a potential non-linear trend for PALS (*p* = 0.073), which was subsequently modeled with a spline in the primary association analysis (Figure 1B). For the final prediction models, predictors were retained in their linear form for simplicity and clinical interpretability, as the non-linearity for PALS was not statistically conclusive and the linear assumption provided a good fit.

To mitigate overfitting, model coefficients were estimated using ridge penalization. The optimal penalty parameter (*λ*) was selected via 10-fold cross-validation on the development dataset, aiming to minimize the penalized partial likelihood. This process was repeated within each of the 1,000 bootstrap samples used for internal validation to obtain optimism-corrected estimates. Penalization was applied uniformly to all three models (Clinical, Clinical + PALS, and Clinical + LAVI) to ensure fair comparison and consistent handling of potential overfitting.

#### 2.9.2 Assessment of PALS association

To demonstrate the independent association of PALS, a separate multivariable Cox proportional hazards model was fitted, adjusting for the complete set of potential confounders, including the 7 clinical variables, LAVI, and LVEF. The proportional hazards assumption was verified using Schoenfeld residuals. No significant violations were detected (all global test *p*-values > 0.05; see Supplementary Table S2 for detailed results).

#### 2.9.3 Model performance and internal validation

Model performance was assessed with discrimination (Harrell's C-index, time-dependent AUC at 12 months), calibration (calibration slope and intercept, calibration plots), and overall accuracy (Brier score). The incremental value of PALS was assessed by comparing the AUC of the Clinical + PALS model with that of the Clinical model using DeLong's test. Internal validation was performed using 1,000 bootstrap samples to calculate optimism-corrected performance estimates for all metrics.

#### 2.9.4 Reclassification and clinical utility

Reclassification improvement was quantified using the continuous Net Reclassification Improvement (NRI) and Integrated Discrimination Improvement (IDI). Category-based NRI was calculated at clinically relevant risk thresholds of 10%, 20%, and 30% (14). Decision curve analysis was used to evaluate the clinical net benefit of the Clinical + PALS model (15). The threshold probability range of 10% to 30% was selected *a priori*, reflecting the spectrum of risk at which clinicians typically consider actionable changes in post-ablation management, such as initiating intensified monitoring or discussing repeat ablation. The net benefit of the Clinical + PALS model was compared to that of the Clinical model and the “treat-all” and “treat-none” strategies across this range.

#### 2.9.5 Risk stratification, sensitivity, and missing data

Using predicted probabilities from the Clinical + PALS model, patients were stratified into low- (<10%), intermediate- (10%–20%), and high-risk (>20%) groups. Observed event rates and actual reablation rates across these strata were compared. Pre-specified sensitivity analyses included: (i) adding LAVI to the primary Clinical + PALS model, (ii) adjusting for post-blanking antiarrhythmic drug use, and (iii) testing for interaction between PALS and the rhythm (sinus vs. AF) during its acquisition. Missing data (<5% for any variable) were handled using Multiple Imputation by Chained Equations (MICE, 20 imputed datasets).

## 3 Results

### 3.1 Study participants and flow

**Figure 2:**
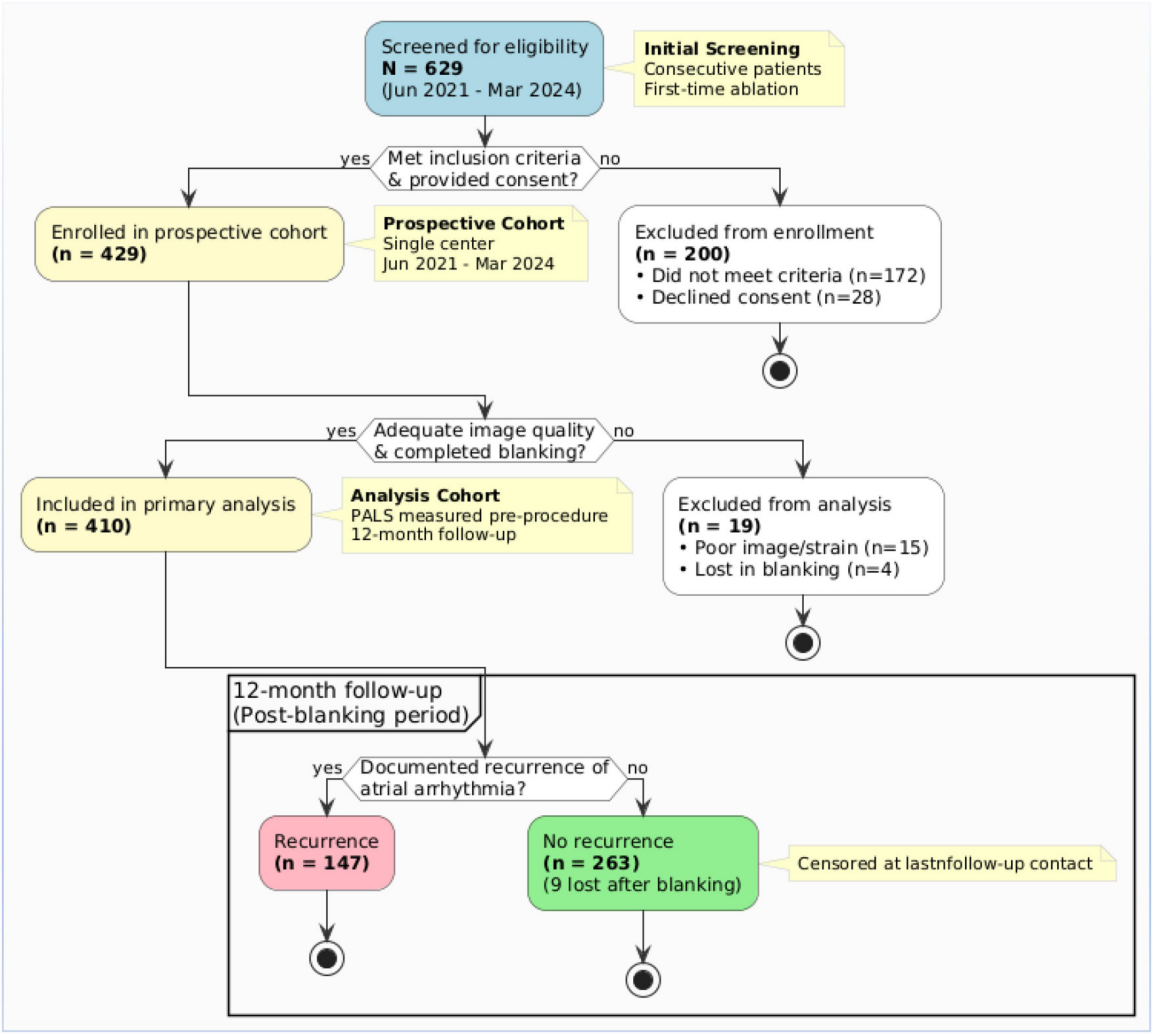
Flowchart of patient screening, enrollment, and follow-up.

Of 629 consecutive patients with persistent atrial fibrillation screened between June 2021 and March 2024, 200 were excluded (172 did not meet eligibility criteria, 28 declined consent). The remaining 429 patients were enrolled in the prospective cohort. After excluding 15 patients due to inadequate pre-procedural image quality and 4 who were lost to follow-up during the 90-day blanking period, 410 patients constituted the primary analysis cohort (Figure 2).

### 3.2 Baseline characteristics and follow-up

Among the 410 analyzed patients, 147 (35.9%) experienced a recurrence of atrial arrhythmia within 12 months after the blanking period. Compared to the non-recurrence group, patients with recurrence were older, had a more extended history of AF, and had higher prevalences of hypertension and diabetes. As expected, the recurrence group had significantly worse left atrial metrics: lower PALS and a higher LAVI (Table 1).

**Table 1:** Baseline characteristics of the analysis cohort (*n* = 410).

Variable	Overall (*n* = 410)	Recurrence (*n* = 147)	No Recurrence (*n* = 263)
Demographics			
Age, years	64 (58, 71)	66 (60, 72)	63 (57, 70)
Sex, male	279 (68.1)	109 (74.2)	170 (64.6)
Clinical History			
Atrial fibrillation duration, years	4.1 (2.0, 7.2)	4.9 (2.8, 8.3)	3.7 (1.8, 6.4)
Body mass index, kg/m²	26.9 (24.6, 29.8)	27.4 (25.1, 30.7)	26.6 (24.3, 29.2)
Hypertension	252 (61.5)	103 (70.1)	149 (56.7)
Diabetes mellitus	86 (21.0)	39 (26.5)	47 (17.9)
History of stroke/TIA	39 (9.5)	18 (12.2)	21 (8.0)
Obstructive sleep apnea	78 (19.0)	34 (23.1)	44 (16.7)
eGFR, mL/min/1.73 m²*	86 (72, 98)	82 (69, 95)	89 (75, 100)
Imaging Metrics			
PALS, global %^†^	19.6 (15.9, 23.3)	17.4 (13.8, 20.9)	21.0 (17.6, 24.8)
PALS, 4-chamber view %	19.1 (15.3, 23.1)	16.7 (13.1, 20.5)	20.5 (17.1, 24.3)
PALS, 2-chamber view %	20.0 (16.1, 23.8)	17.9 (14.2, 21.4)	21.3 (17.8, 25.1)
LAVI, mL/m²^‡^	39 (32, 47)	43 (36, 51)	36 (31, 43)
LVEF, %^§^	60 (55, 64)	58 (53, 62)	61 (56, 65)
Acquisition Details			
Sinus rhythm at acquisition	251 (61.2)	76 (51.7)	175 (66.5)
Heart rate at acquisition, bpm	78 (68, 89)	82 (72, 94)	75 (66, 86)
Frame rate, frames/sec	72 (65, 82)	71 (64, 81)	73 (65, 83)

Data are presented as *n* (%) or Median (Interquartile Range).

* eGFR, estimated glomerular filtration rate, calculated using the CKD-EPI 2021 formula.

† PALS, peak atrial longitudinal strain, measured within 14 days before the index ablation procedure.

‡ LAVI, left atrial volume index.

§ LVEF, left ventricular ejection fraction. TIA, transient ischemic attack.

All procedures included pulmonary vein isolation as the foundational strategy. Adjunctive substrate modification (e.g., posterior wall isolation, linear ablation) was performed at the operator's discretion based on individual patient anatomy and electrophysiological findings. This procedural heterogeneity was accounted for in our modeling framework through a prespecified sensitivity analysis (see Sensitivity Analyses, Section 3.7 and Supplementary Table S6), which confirmed that the independent predictive value of PALS remained stable after adjustment for ablation strategy (HR 1.06, *p* < 0.001).

Follow-up completeness was high, with scheduled Holter completion rates exceeding 89% at all time points, and a post-blanking loss-to-follow-up rate of 2.2% (see Supplementary Table S2 for details).

### 3.3 Reproducibility of PALS measurement

The reproducibility of pre-procedural PALS measurements was excellent. Intraobserver and interobserver intraclass correlation coefficients (ICC) ranged from 0.91–0.93 and 0.87–0.91, respectively, with Bland-Altman biases of less than 0.20% (Supplementary Table S3).

### 3.4 Association of PALS with recurrence risk

A transparent gradient of risk was evident across PALS tertiles, with the lowest tertile associated with the highest recurrence rate (log-rank *χ*^2^ = 30.1, *p* < 0.001) (Figure 1A). Restricted cubic spline analysis confirmed a strong, approximately linear inverse relationship between PALS and recurrence risk (*p* for overall association <0.001; *p* for non-linearity = 0.073) (Figure 1B).

In a multivariable Cox model adjusting for clinical factors, LAVI, and left ventricular ejection fraction, each 1% decrease in PALS remained independently associated with a 6% increase in the hazard of recurrence (HR 1.06, 95% CI 1.03–1.10; *p* < 0.001) (Supplementary Table S4).

### 3.5 Performance of the prediction model

The predictive performance of the model integrating PALS with clinical features (Clinical + PALS) was superior to that of the Clinical-only model. Discrimination improved significantly (C-index: 0.74 vs. 0.66; ΔAUC at 12 months: 0.08, *p* < 0.001). The improvement from adding the structural metric LAVI was marginal (ΔAUC: 0.03, *p* = 0.084) (Table 2).

**Table 2:** Comparison of model performance for predicting 12-month recurrence after catheter ablation.

Metric	Clinical Model	Clinical + PALS Model	Clinical + LAVI Model
Discrimination			
Harrell's C-index (95% CI)	0.66 (0.62, 0.70)	0.74 (0.70, 0.77)	0.69 (0.65, 0.73)
AUC at 12 months (95% CI)*	0.67 (0.63, 0.71)	0.75 (0.71, 0.79)	0.70 (0.66, 0.74)
ΔAUC vs. Clinical (95% CI)	–	0.08 (0.05, 0.11)	0.03 (−0.01, 0.06)
*p*-value (DeLong test)	–	<0.001	0.084
Calibration & Accuracy			
Brier Score at 12 months*	0.21 (0.19, 0.23)	0.18 (0.16, 0.20)	0.20 (0.18, 0.22)
Akaike Information Criterion	1,312	1,271	1,303

* AUC and Brier scores were calculated using inverse probability of censoring weighting (IPCW). The Clinical Model includes the following variables: age, sex, atrial fibrillation duration, body mass index, hypertension, diabetes, and estimated glomerular filtration rate. AUC, area under the receiver operating characteristic curve; LAVI, left atrial volume index; PALS, peak atrial longitudinal strain.

**Figure 3:**
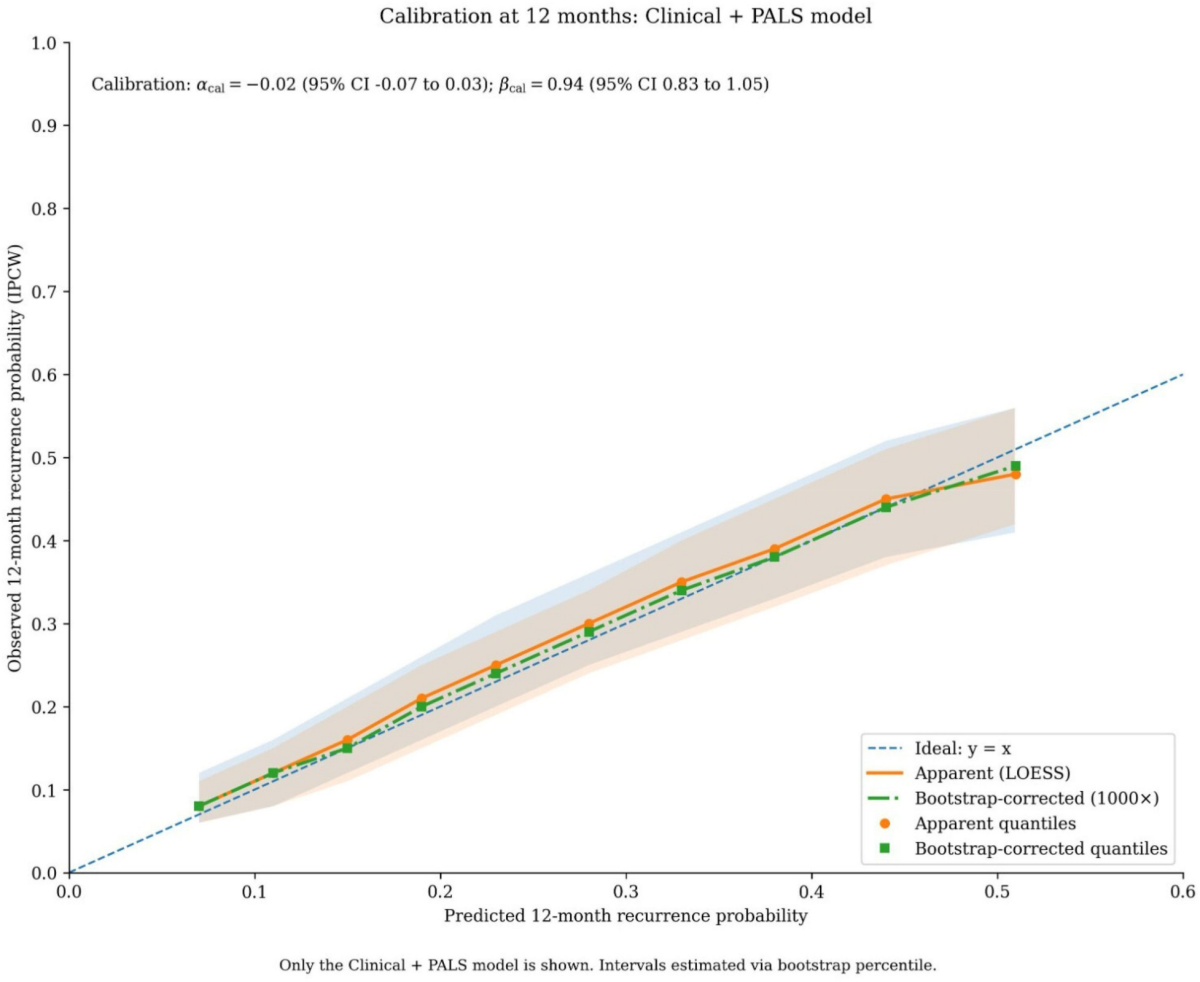
Calibration of the clinical + PALS model for predicting 12-month recurrence risk. Calibration plot assessing the agreement between predicted probabilities of recurrence (*x*-axis) and observed event frequencies (*y*-axis) at the 12-month time point. The diagonal dashed line represents the ideal line of perfect calibration. The solid blue line and shaded band show the observed calibration curve with 95% confidence intervals, estimated using locally weighted scatterplot smoothing (LOESS). The histogram at the top displays the distribution of patients' predicted probabilities. Calibration intercept (*α*) = −0.02; calibration slope (*β*) = 0.94.

The Clinical + PALS model was well-calibrated, with predicted probabilities showing close agreement with observed outcomes across the risk spectrum (Figure 3).

The addition of PALS led to a significant improvement in reclassification. The continuous net reclassification index (NRI) was 0.58 (*p* < 0.001) and the integrated discrimination improvement (IDI) was 0.07 (*p* < 0.001). Categorical NRI was significant across clinically relevant risk thresholds (Supplementary Table S5).

### 3.6 Clinical utility and risk stratification

**Figure 4:**
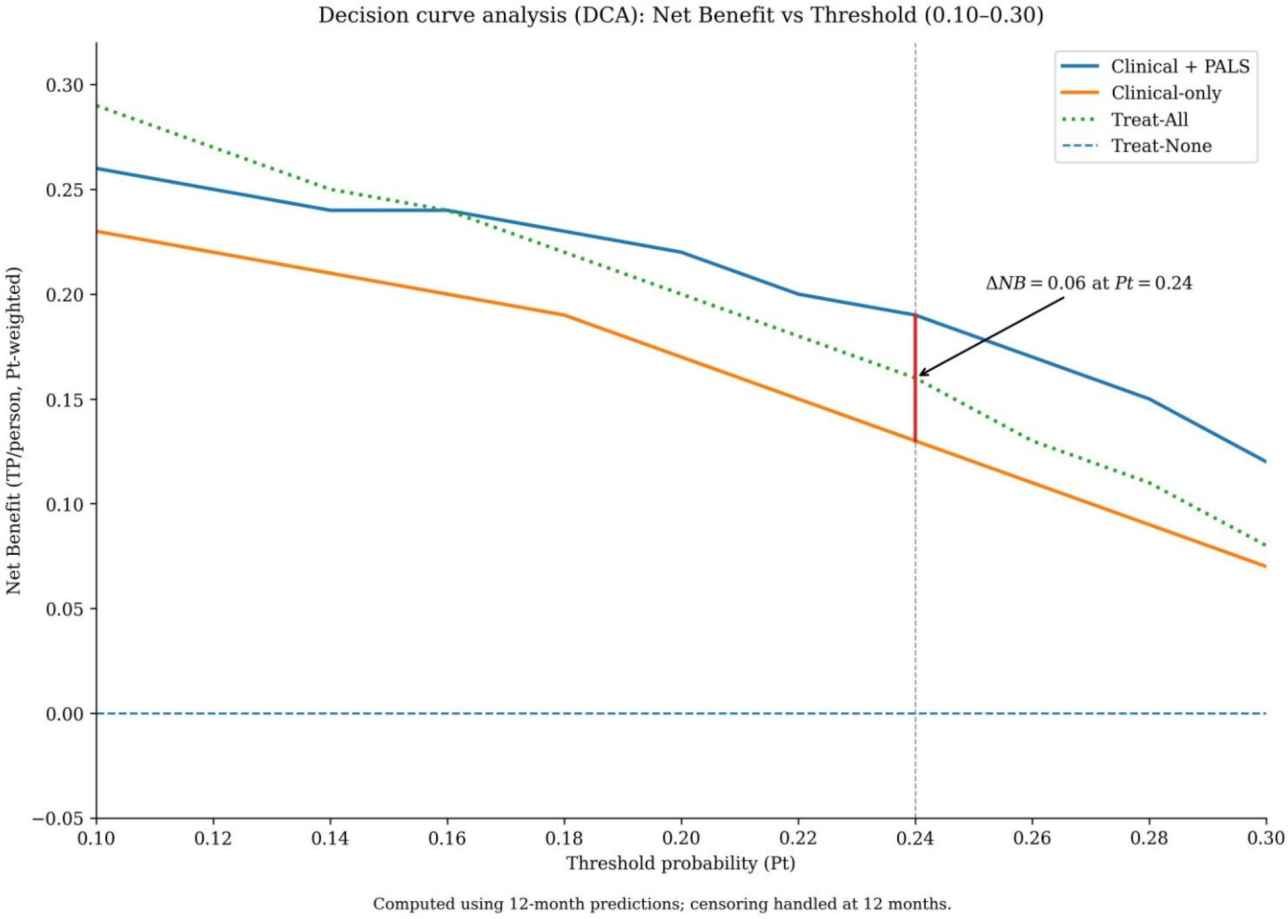
Decision curve analysis evaluating the clinical utility of the prediction models. Net benefit of using the Clinical + PALS model (red solid line), the Clinical-only model (blue dashed line), the “treat-all” strategy (gray solid line), and the “treat-none” strategy (black dashed line) across threshold probabilities from 0.10 to 0.30. The Clinical + PALS model provides the highest net benefit across this clinically relevant range for considering intensified monitoring or repeat ablation. The maximal difference in net benefit from the Clinical-only model (ΔNB = 0.06) occurs at a threshold probability of 0.24.

Decision curve analysis was performed to evaluate the clinical utility of the models across a range of threshold probabilities. The range of 10% to 30% was selected as clinically pertinent, representing the spectrum at which clinicians might consider initiating intensified rhythm monitoring (lower end) or discussing a repeat ablation procedure (higher end) for a patient with persistent AF. Within this range, the Clinical + PALS model provided a greater net benefit than the Clinical-only model (Figure 4). The maximum difference in net benefit (ΔNB = 0.06) occurred at a threshold probability of 24%. This indicates that, at this decision threshold, using the Clinical + PALS model would lead to 6 more beneficial clinical decisions per 100 patients compared to using the clinical-only model, without increasing the rate of unnecessary interventions.

Stratifying patients into low (<10%), intermediate (10%–20%), and high (>20%) risk groups based on the model's predicted probabilities revealed sharply graduated observed recurrence rates (9.7%, 30.2%, and 73.1%, respectively; *p* for trend <0.001) and actual re-ablation rates (Table 3).

**Table 3:** Risk stratification and observed outcomes based on the clinical + PALS model.

Risk Stratum	Sample Size, *n* (%)	Predicted Risk, Median [IQR] (%)	Observed Recurrence Rate, % (95% CI)	Reablation Rate, %*
Low Risk	136 (33.2)	7.9 (6.2, 9.3)	9.7 (6.4, 13.4)	3.68
Intermediate Risk	154 (37.6)	15.8 (12.3, 18.8)	30.2 (23.8, 36.1)	9.09
High Risk	120 (29.3)	28.7 (22.6, 36.9)	73.1 (64.5, 80.3)	24.17
Test for Differences		*χ*² = 82.48, df = 2, *p* < 0.001	*χ*² = 82.48, df = 2, *p* < 0.001	
Test for Trend		Z = 9.12, *p* < 0.001	Z = 9.12, *p* < 0.001	

* Reablation rate: proportion undergoing repeat catheter ablation within 12 months after the index procedure. Risk strata were defined by individualized predicted probabilities from the Clinical + PALS model: Low (<10%), Intermediate (10%–20%), High (>20%). CI, confidence interval; IQR, interquartile range; PALS, peak atrial longitudinal strain.

### 3.7 Sensitivity analyses

Among the 410 patients, 115 (28.0%) continued antiarrhythmic drug therapy beyond the 90-day blanking period. The independent association between PALS and recurrence risk was robust in sensitivity analyses. It persisted after adjusting for ablation strategy or post-blanking antiarrhythmic drug use (HR 1.06 for both). The effect was consistent regardless of whether PALS was measured during sinus rhythm or atrial fibrillation (*p* for interaction = 0.417) (Supplementary Table S6).

## 4 Discussion

This prospective cohort study demonstrates that PALS independently predicts 12-month recurrence risk after first catheter ablation for persistent atrial fibrillation. The association was approximately linear, robust to multivariable adjustment, and insensitive to acquisition rhythm, ablation strategy, or post-blanking antiarrhythmic drug exposure. When integrated with clinical features, PALS significantly improved model discrimination, calibration, and reclassification, providing tangible net benefit for clinical decision-making across reasonable threshold probabilities.

PALS quantifies left atrial reservoir function during ventricular systole, integrating information on chamber compliance, wall stress, and myofiber orientation (16, 17). Reduced PALS reflects restricted atrial deformation, indicating diffuse fibrosis, interstitial remodeling, and electromechanical dysfunction, a substrate that promotes AF persistence and recurrence after ablation. Notably, PALS retained independent predictive value even after adjustment for left atrial volume and left ventricular ejection fraction, confirming that functional impairment captures distinct pathophysiology beyond geometric dilatation (18). This functional metric appears sensitive to early, microscopic components of structural remodeling and maintains its predictive capacity regardless of the atrial rhythm during measurement. Notably, adding PALS provided substantially greater improvement than adding the conventional structural metric LAVI (ΔAUC 0.08 vs. 0.03), underscoring the added value of functional assessment over geometric measurement alone.

Compared to existing prediction models that rely predominantly on clinical variables and left atrial size (3, 19), our PALS-enhanced model offers superior performance. The consistent improvement in discrimination (C-index increase from 0.66 to 0.74), coupled with excellent calibration, represents a meaningful advance. Furthermore, the significant reclassification improvement confirms that PALS meaningfully rearranges patients' risk estimates within clinically relevant ranges (20, 21). While previous studies have often been retrospective, included mixed AF populations, or used arbitrary PALS thresholds (22, 23), our prospective design with standardized monitoring and blinded endpoint adjudication provides more substantial evidence for a continuous dose–response relationship specifically in persistent AF.

Two key findings substantiate the clinical utility of our model. First, decision curve analysis demonstrated superior net benefit across threshold probabilities (10%–30%) pertinent to decisions about intensified monitoring or repeat ablation, with maximum benefit at a 24% threshold. Second, risk stratification using the model's predictions revealed sharp gradients in both observed recurrence rates (9.7%, 30.2%, and 73.1% across low-, intermediate-, and high-risk strata) and actual reablation rates. These gradients provide a practical framework for personalizing post-ablation management, potentially reducing unnecessary interventions in low-risk patients while prioritizing early re-evaluation in high-risk individuals (24).

## 5 Limitations and future directions

Several limitations warrant consideration. First, as a single-center study, our findings require external validation in diverse settings; center-specific practices regarding follow-up intensity and reablation thresholds may influence the detection of outcomes and optimal decision thresholds. Second, although our sample size met requirements for model development, larger cohorts might reveal more minor interaction effects. Third, PALS was analyzed using vendor-specific software; differences in tracking algorithms across platforms may affect absolute values and limit generalizability until cross-vendor standardization is achieved. Fourth, our assessment focused on global left atrial reservoir strain. We did not specifically evaluate the function or morphology of the left atrial appendage (LAA), a known source of triggers and substrate in persistent AF. While global PALS provides a robust measure of the main atrial body's health, future studies incorporating LAA-specific imaging could further refine risk stratification. Fifth, we assessed only preprocedural PALS, not its dynamic changes after ablation or in response to medical therapy. Sixth, our endpoint captured recurrence despite ongoing medical management in a substantial subset of patients (28.0% continued AADs post-blanking), reflecting real-world practice where therapy is often tailored to perceived risk. Our sensitivity analysis confirmed that the predictive value of PALS was robust to adjustment for post-blanking AAD use (HR 1.06, *p* < 0.001), supporting its utility in the common clinical scenario where the decision to continue or withdraw AADs is itself informed by pre-procedural risk assessment. Seventh, while we systematically adjudicated symptomatic and asymptomatic recurrences through protocolized monitoring, some asymptomatic episodes may have been missed. Finally, despite adjusting for key covariates, residual confounding cannot be excluded in an observational study.

Future research should focus on multicenter external validation and recalibration of our model, establishing cross-vendor reference values for PALS, conducting a direct comparison with late gadolinium enhancement cardiac magnetic resonance as a fibrosis reference standard, and investigating dynamic PALS changes post-ablation. Ultimately, prospective trials evaluating model-guided management strategies are needed to determine whether PALS-based risk stratification translates into improved patient outcomes and resource utilization.

## 6 Conclusion

PALS independently and approximately linearly predicts 12-month recurrence risk after first catheter ablation for persistent atrial fibrillation. Integration of PALS with clinical features yields a well-calibrated prediction model with superior discrimination and reclassification compared to models based solely on clinical features or structure-based (LAVI) models. This model provides tangible net benefit for clinical decisions across reasonable risk thresholds and enables stratification into distinct risk categories with sharply differing recurrence and reablation rates. With its excellent measurement reproducibility and robustness across acquisition rhythms and procedural variations, PALS warrants consideration as a core functional metric in standardized preprocedural risk assessment for patients with persistent AF undergoing first ablation.

## Data Availability

The original contributions presented in the study are included in the article/Supplementary Material, further inquiries can be directed to the corresponding author.
