# Supplementary material for: Pre-procedural left atrial strain and clinical features for predicting recurrence after first catheter ablation of persistent atrial fibrillation: a prospective cohort study
Source: Front Cardiovasc Med. 2026 Feb 9;13:1764723. doi: 10.3389/fcvm.2026.1764723 (PMC12926841; doi:10.3389/fcvm.2026.1764723)
Supplement: Supplementary file 1 [file Datasheet1.docx]

**Pre-procedural Left Atrial Strain and Clinical Features for Predicting Recurrence after First Catheter Ablation of Persistent Atrial Fibrillation: A Prospective Cohort Study**

**Authors:** Qunfang Tu**^1^**, and Dongping Xiao**^2^***

**Address:**

**^1^** Department of Emergency, Nanchang First Hospital, Nanchang City, 330000, Jiangxi Province, China.

**^2^** Department of Cardiology, Nanchang First Hospital, Nanchang City 330000, Jiangxi Province, China.

**Correspondence: ***Dongping Xiao**^2^**, **E-mail:** dpxiao@ldy.edu.rs

**Co-Authors:** Qunfang Tu**^1^**, E-mail: tuqunf@163.com

**Table S1**. Detailed reasons for exclusion during initial screening (n=172).

| **Exclusion Category** | **Number** |
| --- | --- |
| Prior left atrial catheter ablation or surgical Maze procedure | 71 |
| Cardiac surgery or percutaneous coronary intervention within preceding 3 months | 22 |
| Significant rheumatic mitral valve disease or mechanical prosthetic valve | 18 |
| Active myocarditis or restrictive/infiltrative cardiomyopathy | 8 |
| Complex congenital heart disease | 6 |
| Age <18 or >85 years | 17 |
| Pregnancy | 4 |
| Expected survival <1 year | 11 |
| Poor acoustic window precluding reliable left atrial strain assessment | 15 |
| **Total** | **172** |

**Table S2.** Testing the proportional hazards assumption for Key Cox Regression Models.

| **Model** | **Schoenfeld Residual Test (Global)** | **χ²** | **df** | **p-value** | **Evidence of Violation?** |
| --- | --- | --- | --- | --- | --- |
| **Primary Association Model** (PALS + Clinical + LAVI + LVEF) | Global Test | 7.23 | 10 | 0.705 | No |
| *– PALS* | Individual Test | 0.12 | 1 | 0.732 | No |
| *– LAVI* | Individual Test | 0.88 | 1 | 0.348 | No |
| *– AF Duration* | Individual Test | 2.45 | 1 | 0.118 | No |
| **Clinical + PALS Prediction Model** | Global Test | 5.84 | 8 | 0.666 | No |
| **Clinical-only Prediction Model** | Global Test | 6.12 | 7 | 0.526 | No |
| **Clinical + LAVI Prediction Model** | Global Test | 5.91 | 8 | 0.657 | No |

**Note:** The assumption of proportional hazards was assessed using Schoenfeld residuals. A statistically significant test (p < 0.05) would indicate a violation of the assumption. As shown, no significant violations were detected for any model or key predictor (all p-values > 0.05), supporting the validity of the Cox proportional hazards models used.

**Table S2.** Follow-up completeness and event ascertainment.

| **Category** | **Metric** | **Overall, n=410** | **Recurrence, n=147** | **No recurrence, n=263** |
| --- | --- | --- | --- | --- |
| **Scheduled Monitoring Adherence** | 3-month 24-hour Holter completed | 382 (93.2) | 138 (93.9) | 244 (92.8) |
|  | 6-month 24-hour Holter completed | 374 (91.2) | 134 (91.2) | 240 (91.3) |
|  | 12-month 24-hour Holter completed | 366 (89.3) | 127 (86.4) | 239 (90.9) |
| **Symptomatic Monitoring** | Symptom-triggered ECG obtained | 196 (47.8) | 97 (66.0) | 99 (37.6) |
| **Device Interrogation** | Remote monitoring activated* | 52 (12.7) | 20 (13.6) | 32 (12.2) |
| **Follow-up Status** | Lost to follow-up (post-blanking) † | 9 (2.2) | 2 (1.4) | 7 (2.7) |
| **Time at Risk** | Evaluable follow-up time, days‡  Median [IQR]  95% CI for median | 273 [261, 276]  (270, 276) | 271 [254, 275]  (266, 275) | 274 [265, 276]  (272, 276) |
| **Sources of Documented Recurrence§** | 12-lead electrocardiogram | – | 48 (32.7) | – |
|  | 24-hour Holter monitor | – | 52 (35.4) | – |
|  | Patch or event recorder | – | 31 (21.1) | – |
|  | Implanted device telemetry | – | 16 (10.9) | – |

Data are presented as number (percentage) for categorical variables and median [interquartile range] for continuous variables. Applies only to patients with implanted cardiac electronic devices (pacemaker, implantable cardioverter-defibrillator, or implantable loop recorder). † Defined as withdrawal or loss of contact after the 90-day blanking period; four additional patients lost during the blanking period were excluded from analysis. ‡ Measured from the end of the blanking period (Index + 90 days) to recurrence, last contact, or censoring at 12 months; the 95% confidence interval (CI) for the median was estimated using the reverse Kaplan–Meier method. § Documentation source for the first recurrence among the 147 patients with events; percentages are relative to the recurrence group.

**Table S3.** Reproducibility of pre-procedural peak atrial longitudinal strain (PALS) Measurements.

| **Indicator** | **Intraobserver** | | **Interobserver** | | **n** |
| --- | --- | --- | --- | --- | --- |
|  | **ICC（95%CI）** | **Bland-Altman Bias (%), LoA*** | **ICC（95% CI）** | **Bland-Altman Bias (%), LoA*** |  |
| PALS global | 0.93 (0.89, 0.96) | 0.07 (−2.56, 2.72) | 0.91 (0.86, 0.94) | −0.14 (−2.96, 2.69) | 49 |
| A4C_PALS | 0.92 (0.88, 0.95) | 0.12 (−2.81, 3.05) | 0.89 (0.84, 0.93) | −0.19 (−3.18, 2.80) | 50 |
| A2C_PALS | 0.91 (0.86, 0.94) | 0.06 (−2.74, 2.84) | 0.87 (0.80, 0.91) | −0.12 (−3.29, 3.07) | 49 |

*LoA: 95% Limits of Agreement. A4C: Apical four-chamber view; A2C: Apical two-chamber view; ICC: intraclass correlation coefficient.

**Table S4.** Multivariable association of pre-procedural pals with 12-month recurrence risk.

| **Variable** | **HR (95% CI)** | **Wald Z** | **p value** |
| --- | --- | --- | --- |
| PALS global (per 1% decrease) | 1.06 (1.03, 1.10) | 3.674 | <0.001 |
| Age (per 1 year) | 1.01 (0.99, 1.03) | 1.070 | 0.285 |
| Sex (male vs. female) | 1.19 (0.89, 1.59) | 1.122 | 0.262 |
| Atrial fibrillation duration (per 1 year) | 1.05 (1.02, 1.08) | 3.424 | 0.001 |
| Body mass index (per 1 kg/m²) | 1.02 (0.99, 1.06) | 1.358 | 0.175 |
| Hypertension (yes vs. no) | 1.24 (0.95, 1.63) | 1.575 | 0.115 |
| Diabetes (yes vs. no) | 1.33 (0.98, 1.80) | 1.862 | 0.062 |
| eGFR (per 5 mL/min/1.73m²) † | 0.96 (0.92, 1.00) | −1.890 | 0.059 |
| LAVI (per 5 mL/m²) ‡ | 1.13 (1.05, 1.22) | 3.147 | 0.002 |
| LVEF (per 5%) § | 0.94 (0.87, 1.01) | −1.781 | 0.075 |

*Cox proportional hazards model. Overall model likelihood ratio χ² = 47.592, p < 0.001. †eGFR, estimated glomerular filtration rate (CKD-EPI 2021 formula). ‡LAVI, left atrial volume index. §LVEF, left ventricular ejection fraction. CI: confidence interval; HR, hazard ratio; PALS: peak atrial longitudinal strain.

**Table S5**. Detailed reclassification improvement after adding PALS to the clinical model.

| **Indicator** | **Point Estimate** | **95% CI** | **p-value** |
| --- | --- | --- | --- |
| **Categorical NRI (Threshold = 10%)** | | | |
| NRI for Events | 0.31 | 0.21 – 0.41 | <0.001 |
| NRI for Non-events | 0.12 | 0.04 – 0.20 | 0.003 |
| Total NRI | 0.43 | 0.30 – 0.56 | <0.001 |
| **Categorical NRI (Threshold = 20%)** | | | |
| NRI for Events | 0.24 | 0.14 – 0.34 | <0.001 |
| NRI for Non-events | 0.11 | 0.03 – 0.19 | 0.006 |
| Total NRI | 0.35 | 0.22 – 0.48 | <0.001 |
| **Categorical NRI (Threshold = 30%)** | | | |
| NRI for Events | 0.19 | 0.09 – 0.29 | 0.001 |
| NRI for Non-events | 0.10 | 0.02 – 0.19 | 0.013 |
| Total NRI | 0.29 | 0.16 – 0.41 | 0.002 |
| Continuous NRI | 0.58 | 0.41 – 0.73 | <0.001 |
| Integrated Discrimination Improvement (IDI) | 0.07 | 0.05 – 0.10 | <0.001 |

Note: Event was defined as recurrence of atrial arrhythmia occurring from the end of the 90-day blanking period to 12 months. The Clinical Model includes age, sex, atrial fibrillation duration, body mass index, hypertension, diabetes, and estimated glomerular filtration rate. NRI, Net Reclassification Improvement; CI, Confidence Interval.

**Table S6.** Sensitivity analyses of the association between pals and recurrence risk.

| **Analysis Scenario** | **Hazard Ratio (95% CI) *** | **Wald Z** | **p-value** | **p for Interaction** |
| --- | --- | --- | --- | --- |
| **Adjustment for Procedural Factors** | | | | |
| Main model† + ablation strategy‡ | 1.06 (1.03, 1.10) | 3.529 | <0.001 | — |
| Main model† + post-blanking AAD use§ | 1.06 (1.03, 1.09) | 3.658 | <0.001 | — |
| **Effect Modification by Acquisition Rhythm** | | | | |
| Subgroup: Sinus rhythm at acquisition | 1.05 (1.01, 1.09) | 2.481 | 0.013 | 0.417 |
| Subgroup: Atrial fibrillation at acquisition | 1.07 (1.03, 1.12) | 3.247 | 0.001 |  |
| **Alternative Structural Predictor** | | | | |
| Model with LAVI instead of PALS (per 5 mL/m²) | 1.14 (1.06, 1.23) | 3.221 | 0.001 | — |

*Hazard ratio for PALS expressed per 1% decrease, unless otherwise specified. †The "main model" adjusts for: age, sex, atrial fibrillation duration, body mass index, hypertension, diabetes, estimated glomerular filtration rate, left atrial volume index, and left ventricular ejection fraction. ‡Ablation strategy was included as a categorical covariate (pulmonary vein isolation only vs. extended ablation). § post-blanking antiarrhythmic drug (AAD) use: binary covariate indicating continued AAD use after the 90-day blanking period. AAD: Antiarrhythmic drug; CI: Confidence interval; LAVI: Left atrial volume index; PALS: Peak atrial longitudinal strain.
